# mRNA Detection with Fluorescence-base Imaging Techniques for Arthritis Diagnosis

**Published:** 2019-07-16

**Authors:** Anne Yau, Hongchuan Yu, Yupeng Chen

**Affiliations:** 1 Department of Biomedical Engineering, University of Connecticut, Storrs, CT 06269, USA; 2 Department of Orthopedics and Department of Molecular Pharmacology, Physiology and Biotechnology, Brown University, Providence, RI 02903, USA; 3 Dicerna Pharmaceuticals, Cambridge, MA 02140, USA

**Keywords:** mRNA detection, Fluorescence, Diagnosis, Arthritis

## INTRODUCTION

Arthritis is a term used to indicate joint pain that are caused by inflammation of joints or joint disease that occur commonly among older people. People of all ages, sexes and race can and do have arthritis as well. There are different types of arthritis with different causes and treatment methods but the most commonly known are the Rheumatoid Arthritis (RA) and the Osteoarthritis (OA). The symptoms of arthritis are joint pain, swelling, stiffness and decreased range of motion which could develop over time or appear suddenly. These symptoms may or may not exacerbate over time. As the disease get more severe, it could prevent patients from doing daily activities such as walking up the stairs and experience chronic pain [1].

There are multiple approaches for diagnosing arthritis other than performing physical examinations by the doctor or rheumatologists. Conventional radiography (CR) is one of the many imaging methods for diagnosis. CR is cheap compared to the alternative, magnetic resonance imaging (MRI) and faster than ultrasound. However, CR can only be used towards the end of the disease process, where the bones have eroded and joint spaces narrowing [1] and it does not show soft tissue sufficiently [2]. Both ultrasound and MRI are not favorable due to the time it takes for diagnosis with ultrasound and the high cost in exchange for high sensitivity of MRI. Positron emission tomography (PET) has made its way into diagnosing the disease for its high sensitivity detection and potential for whole-body evaluation [3]. However, due to the short half-life of some isotopes used for this technique, patients will mostly be subjected to radiation exposure which may cause more health problems in the future. Optical imaging (OI) have been around in the microscopic world is now making its way to the macroscopic setting where this technique offers high sensitivity for detection of inflammation [4,5], fast and inexpensive and most importantly do not expose patients to ionizing radiation [3]. OI is perfect for detecting and diagnosing rheumatoid arthritis that mainly affects small joints of hands and feet. Many medical equipment, such as the X-ray radiography, computed tomography (X-RAY CT), radionuclide imaging using single photons (Single Photon Emission Computer Tomography SPECT) and positrons (Positron Emission Tomography PET), magnetic resonance imaging (MRI), ultrasonography (US) and optical imaging require ‘contrast agents’ for the extraction of information of the human bodies [6,7]. Real-time PCR, fluorescence *in situ* hybridization (FISH) analysis have been widely used at the cellular level to detect expression levels and cell distribution of mRNA [8].

Examples of contrast agents are the super paramagnetic or paramagnetic metals, such as Gadolinium Contrast Medium, used in MRI that mainly observed the whole-body scan. To get a more target specific imaging, molecular imaging such as PET and optical imaging like fluorescence microscopy are used for their sensitivity and specificity for target detection. However, the disadvantages uses of PET are that they have poor spatial and temporal resolution. They also require radioactive compounds that have intrinsically limited half-life which limit their repeated use due to safety regulation [9,10]. In this review, we will focus on detecting mRNA at real-time with fluorescence microscopy and the diagnosis of arthritis with fluorescence-based imaging techniques.

## FLUORESCENCE-BASED IMAGING TECHNIQUES

In the past few centuries, there was a surge in the use of fluorescent dye in various areas. Special dyes are used for printing valuable documents such as money, and certificates. Some dyes are used to aid in leak detections of oils and fluids in vehicles. Because of its distinct ability, the dyes are also used to assist in exploration of water underground. Using similar concept in large scale, researchers started to apply this approach in *in vivo* medical imaging. Despite countless of organic dyes in the market for research, there are only two fluorophores that’s approved by the US Food and Drug Administration (FDA) for medical use, which are the fluorophores are indocyanine green (ICG) and fluorescein [11–13]. ICG is used as an ophthalmologic agent and as a hepatic functional agent while fluorescein is used primarily in ophthalmology. These two agents, with no toxicity reported, are primarily used to obtained retinal angiograms that require high dosage of the agents. Even though fluorophores dyes in optical imaging have made great strides in the imaging field, an ideal labeling reagent would be the ones that remain non-fluorescent until bound to its target.

Fluorescence microscopy, providing real-time visualization in the surgical field, has similar sensitivity to radionuclide imaging, providing high resolution, high contrast, high specificity and quantitative of the sample. The downside of optical imaging is that uncertainty emerges as the scans get into deep tissue due to unpredictable light scattering and absorption [14,15]. Hence, the best place to use optical imaging is on the superficial tissue surfaces such as the breasts or the lymph nodes [16–20]. However, with the continued growth in the medical imaging techniques, dyes used for contrast images have improved significantly.

To obtain a successful optical molecular probe for medical imaging, the right wavelength must be employed to excite the dye to obtain a unique emission wavelength. However, if the excitation wavelength is near the ultraviolet region, the tissue will be damaged, while excitation on at the infrared region will cause tissue heating. Absorbance and auto fluorescence could be observed in any case when there is asymmetrical stokes shift [12]. To prevent the auto fluorescence by the tissue under low interference, many researchers and scientists have started applying light in the near-infra red (NIR) wavelength (650–900 nm) as the excitation energy. These NIR wavelengths are invisible to human eyes [21]. The incorporation of NIR fluorophores with current optical imaging techniques provides an intraoperative image-guided surgery that allows for target-specific imaging due to ultralow background auto fluorescence [15].

Previous study of OI have shown promising results after injection of fluorescent dyes for detection of arthritis [4,5,22,23]. Another study has successfully shown that *in vivo* leukocyte cell can be tracked by using optical imaging techniques. These *in vivo* leukocyte cells are labeled with fluorescent dyes, which are suitable for the detection of synovial inflammation in an antigen-induced arthritis model [3].

There are few available fluorescence-based imaging techniques: fluorescence microscopy, flow cytometry, cell sorting fluorescence correlation spectroscopy as well as particle tracking velocimetry [7].

### Visualization mRNA with different hybridization methods

#### FISH

**Figure 1.**
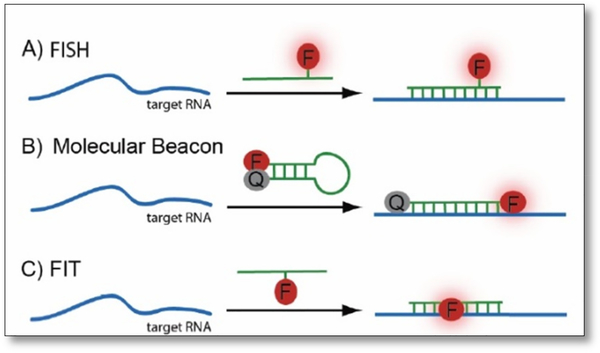
Three different hybridization techniques. (A) Fluorescence *in situ* hybridization (FISH) consists of a DNA strand analogous to its target together with a fluorescence dye. (B) Molecular beacon (MB) is a hairpin like structure that has a quencher on the 3’ end and reporter dye on the 5’. When it reaches the target, the hairpin opened, separating the quencher and dye enabling fluorescence. (C) Forced intercalation (FIT). The fluorophores with DNA strand similar to its target is only enabled when it binds with the target.

The localization of mRNA in cell has been around since 1980s, but then, mRNA translation could only be visualized via *in situ* hybridization (ISH) using probes with multiple fluorophores or multiple probes with one fluorophore [24]. The groups have succeeded in transcript detection *in situ*, enabling genotyping of individual transcript molecule. The earliest single molecular RNA imaging studies was transcribed and fluorescently labeled *in vitro* [25]. As seen in Figure 1A, hybridization-based methods for RNA imaging are as shown. Fluorescence *in situ* Hybridization (FISH) can differentiate RNA molecules with just a single base because it only binds to specific parts of the nucleic acid. FISH are convenient procedures due to its highly sequence-specific when combined with amplifications during procedures in fixed cells [26,27]. Not only FISH can detect and localized RNA targets in cells, cancer cells and tissues, it also assists in defining dimensions of gene expression in cells and tissues in addition to exploring cell reproduction cycle. The fluorophores are usually attached to a strand analogous to its target and in FISH, the progress of the probes can be observed from the very beginning to the end, when it reaches its target, because of its fluorescence nature. It is also important to note that the probe size must be small enough to complement the target, and that multiple colored probes (with different wavelengths) can be used together.

#### Molecular beacons

**Figure 2.**
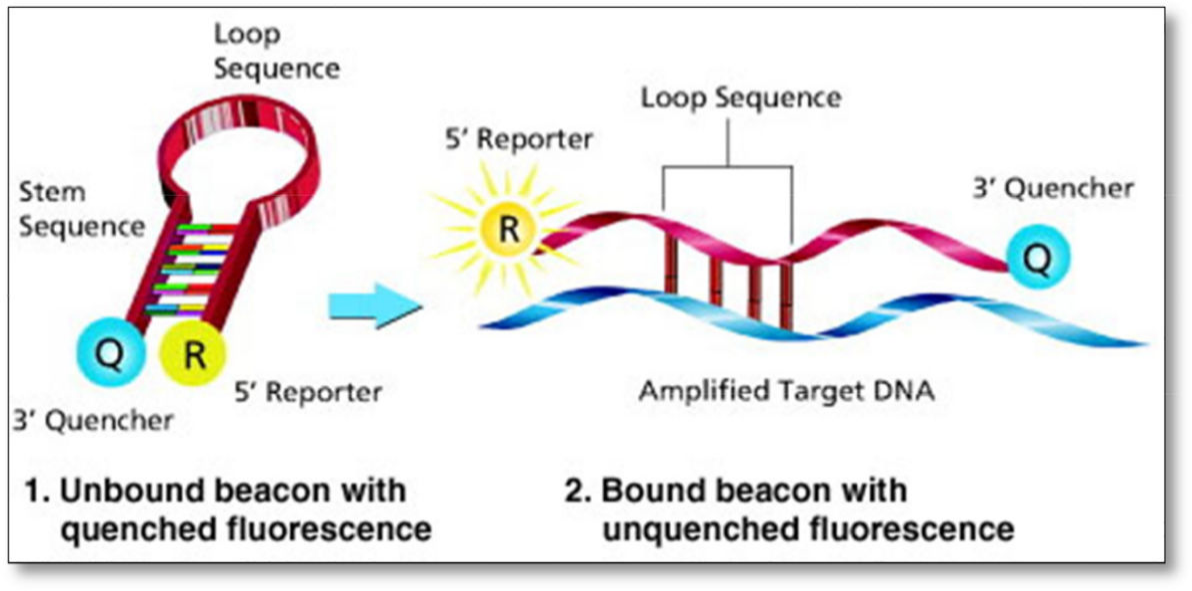
Molecular beacons used in applications involving real-time mRNA detection in living cells. The hair-pin like structure is equipped with 3’quencher and 5’reporter which is an organic dye. Once the beacon reaches target, it will open up, enabling fluorescence.

Molecular beacons (MB) have been used in variety of applications involving real-time mRNA detection in living cells [28], DNA-RNA hybridization studies and protein/DNA interactions [29]. MB, as seen in Figure 2, is a hairpin-like oligonucleotides probe consisted of single-stranded DNA molecule in a stem-loop conformation with a fluorophore linked to 5’ end and a quencher at the 3’ end with a minimal distance from each other. When the molecular beacon reaches its target, the hairpin-like structure then stretches out, separating the fluorophore and quencher, enabling fluorescence, with about 100 times more intensely than background levels of unbound probes.

An example for the success of molecular beacon is explained in a paper published in 2011 by Bratu et al. [29]. Molecular beacons in the paper are synthesized from modified nucleic acids (2’-O-methyl RNA and DNA) to increase target specificity and sensitivity and labeled with various fluorophores and specific quenchers respectively as seen in Tables 1 and 2. They concluded that they were able to synthesize small MB from 2’-O-methyl RNA/LNA chimeric nucleic acids and these hairpins are observed to be stable in cellular environment and high affinity for binding to target RNAs. They also demonstrated that different fluorophores can be used to make the tiny MB which aided in detecting highly structured RNAs, small RNAs and microRNAs which can be seen in Tables 1 and 2.

**Figure 3.**
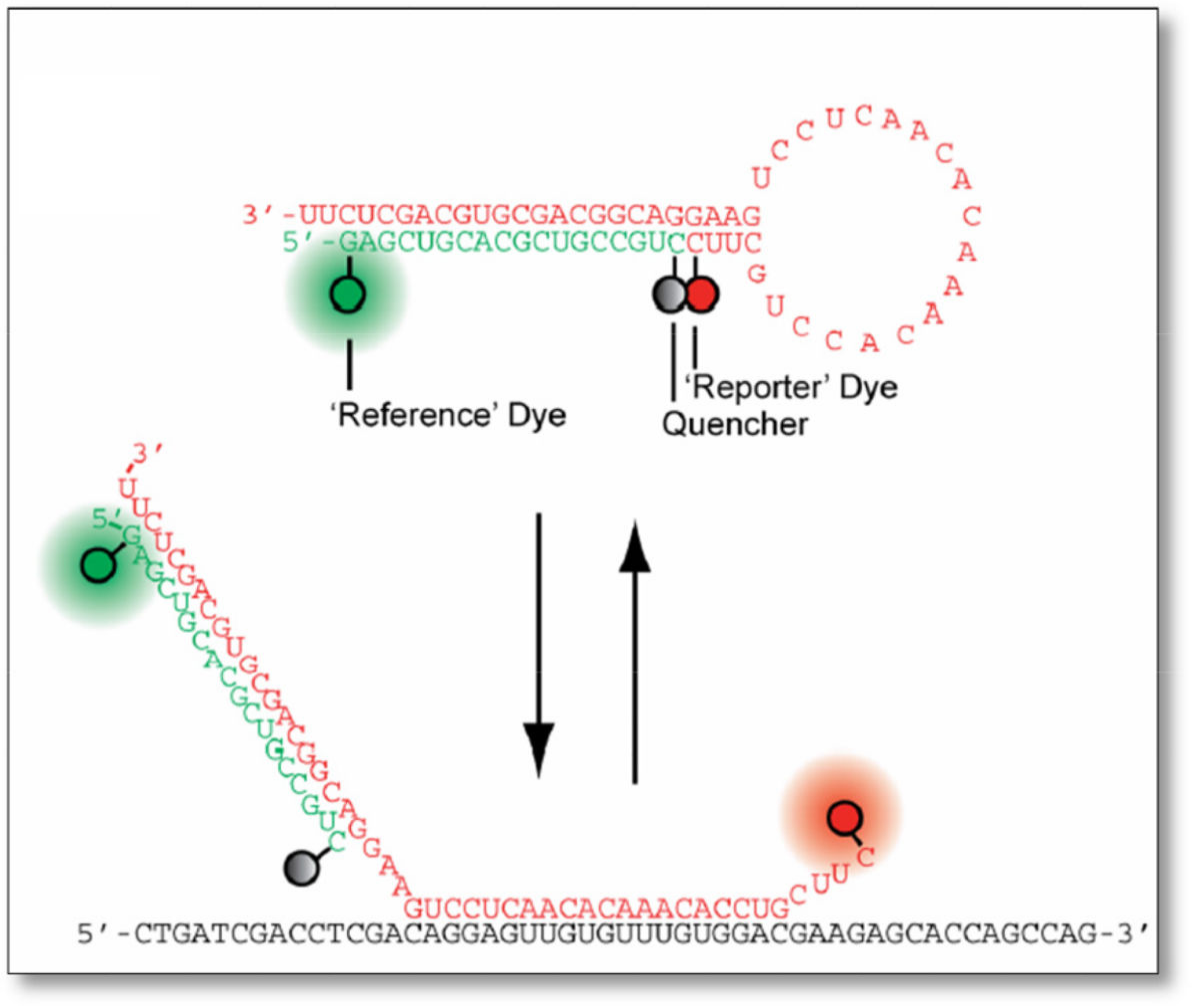
Ratiometric Biomolecular Beacon (RBMB). The reference dye is “on”, while the ‘reporter’ dye is quenched before reaching the target. Upon hybridization, reporter dye and quencher will separate, showing two sets of fluorescence indicating success in hybridization.

Upon the success of molecular beacons for fluorescence optical imaging, another research group have developed ratiometric bimolecular beacons (RBMBs), to scan the directed transport of single engineered RNA transcripts in living cells in real-time in 2013. These RBMBs are designed to overcome the hurdles posed by conventional MBs and to improve signal-to-background ratio [30]. When unbound to the target, RBMBs are in a resting state, which is the hairpin structure like MBs. However, RBMBs do not have fluorophore in the 5’ end and quencher in the 3’ end. As seen in Figure 3, an extra unquenched reference dye was introduced in the 5’ end of the 18-base pair double-stranded domain beacon leaving the 3’UU end unhinges. The fluorophore and quencher are right next to each other, the uncertainty of the distances between fluorophores and quenchers can be eliminated in this design.

This design was hypothesized to reduce level of false-positive detected for 24 h compared to the conventional MBs. The unquenched reference dye is acting like a control to allow measurements of the reporter to be adjusted for differences in RBMBs delivery leading to more precise measurement of RNA hybridization. Their conclusion in this study that RBMBs may be the tools to analyze single engineered RNA transcripts in living cells.

#### FIT

A different approach to increase fluorescent signal upon bonding is to use forced intercalation (FIT) probes as seen in Figure 1C. Like FISH, FIT probes are made of peptide nucleic acid (PNA) or DNA single strands that only fluoresce as soon as it hits the target DNA [31,32]. This is not covered in the review.

#### QUANTUM DOTS

Quantum dots (QD) are a new class of fluorescent probes starting to emerge in the past few decades. QDs also have long lifetime increasing the probability of adsorption and producing broad absorption spectrum. Other than being non-toxic to samples, one of the most appreciated advantages of QD is their photostability or resistance to photobleaching, which allows images to be recorded over a longer period compared to conventional fluorescent dyes [7]. Other than *in vivo* targeting and imaging, QDs are also used in cellular targeting and imaging, as well as fixed tissue analysis, optical encoding and quantitative determination [33].

Size of the quantum dot nanoparticles in bioimaging field generally falls within 2–10 nm in diameter. QDs have unique optical and electronic properties, with molar extinction coefficients that are 10–50 times larger, making it much brighter than conventional dyes. The emission wavelengths are size tunable depending on the radius of the QD. Larger QDs were also synthesized to be used in other applications other than *in vivo* imaging. However, parameters such as the distance between gold nanoparticle to optical absorbance and scattering intensity of the inter-particle distance can be controlled, resulting in a research group utilizing 20 nm Au nanoparticles QD to predict the localized surface plasmon resonance (LSPR) of gold nanoparticles. They were able to synthesize 20 nm AuNP monomers with zero LSPR background as probes with only a single target molecule required to form a dimer with significant plasmon resonance coupling effect, increasing the sensitivity of the LSPR sensor to single cell level. In their study, they were able to present a smart single mRNA imaging approach in living cells based on target-induced formation of nanoparticle dimers. They concluded that with proper linker, these nanoparticles could serve as a basis to many imaging techniques for biomolecules and mRNA in living cells [34].

**Figure 4.**
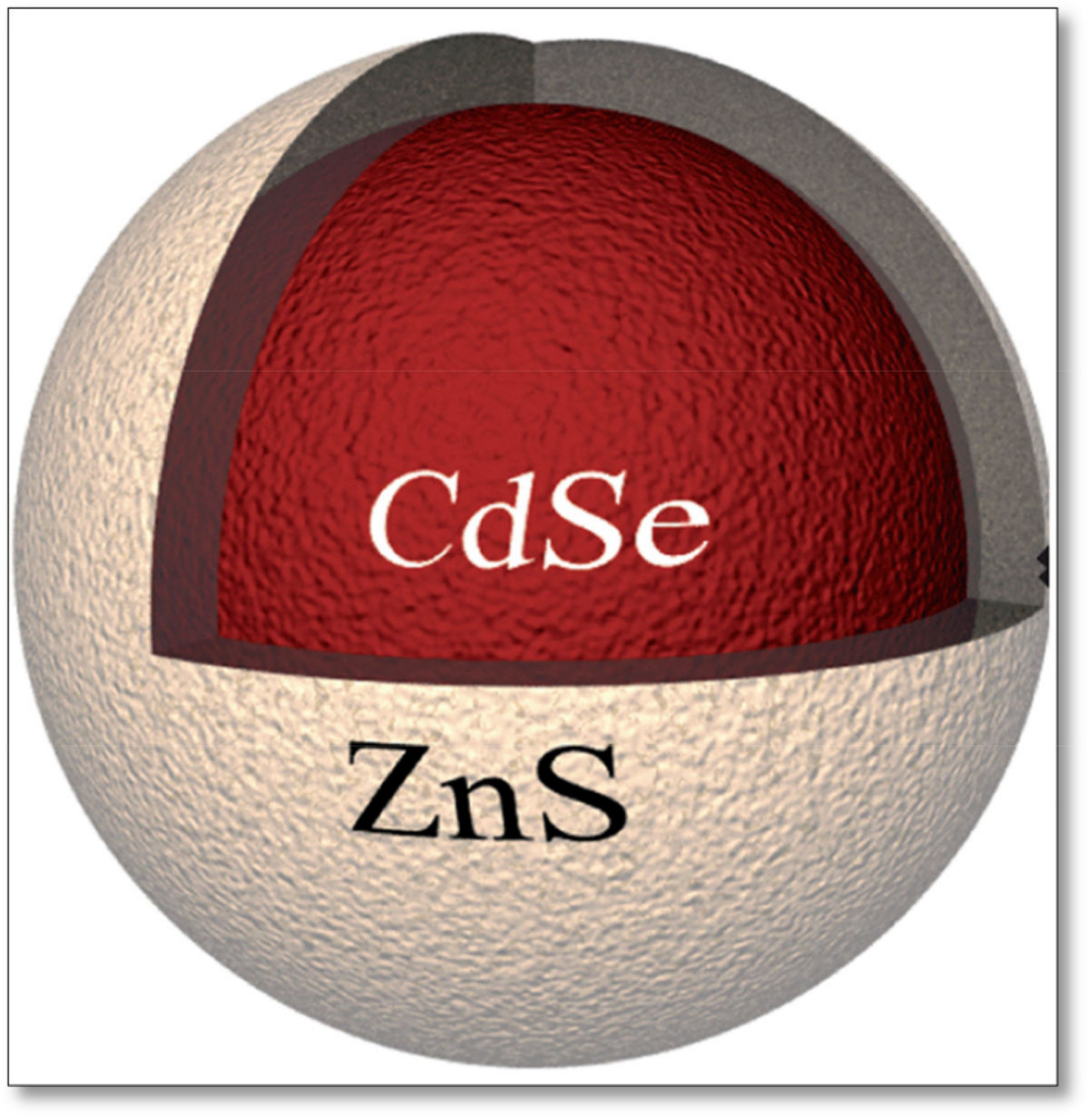
Quantum Dots of CdSe core with ZnS shell.

The optical properties of quantum dots originated from their elemental composition, with a semiconductor core such as cadmium selenide (CdSe) or lead selenide (PbSe), coated with a semiconductor shell, as seen in Figure 4. Properties of QDs have made an impression in the optical imaging field, prompted a topic of intensive interest in cancer biology, molecular imaging and molecular profiling [32,35–42]. Although QDs presented the researchers numerous advantages over traditional organic dyes, these inorganic semiconductor materials are toxic to living systems, limiting their use in biological systems, triggering a hot topic for research in the field on synthesizing a more biocompatible QD for the biological systems. Multiples experiments have been conducted on modified QDs, such as extra surface coating to minimize the cytotoxicity [32]. For example, cadmium ions present in the many QDs are shown to bind to thiol groups on important molecules in the mitochondria causing cell death [32]. The cadmium ions present in the cells are most likely caused by QD exposure to air and UV light, causing the surface to oxidize in oxidative solution. The levels of cytotoxicity could be reduced or eliminated by addition surface coatings [32].

#### QUANTUM DOTS and MOLECULAR BEACONS

**Figure 5.**
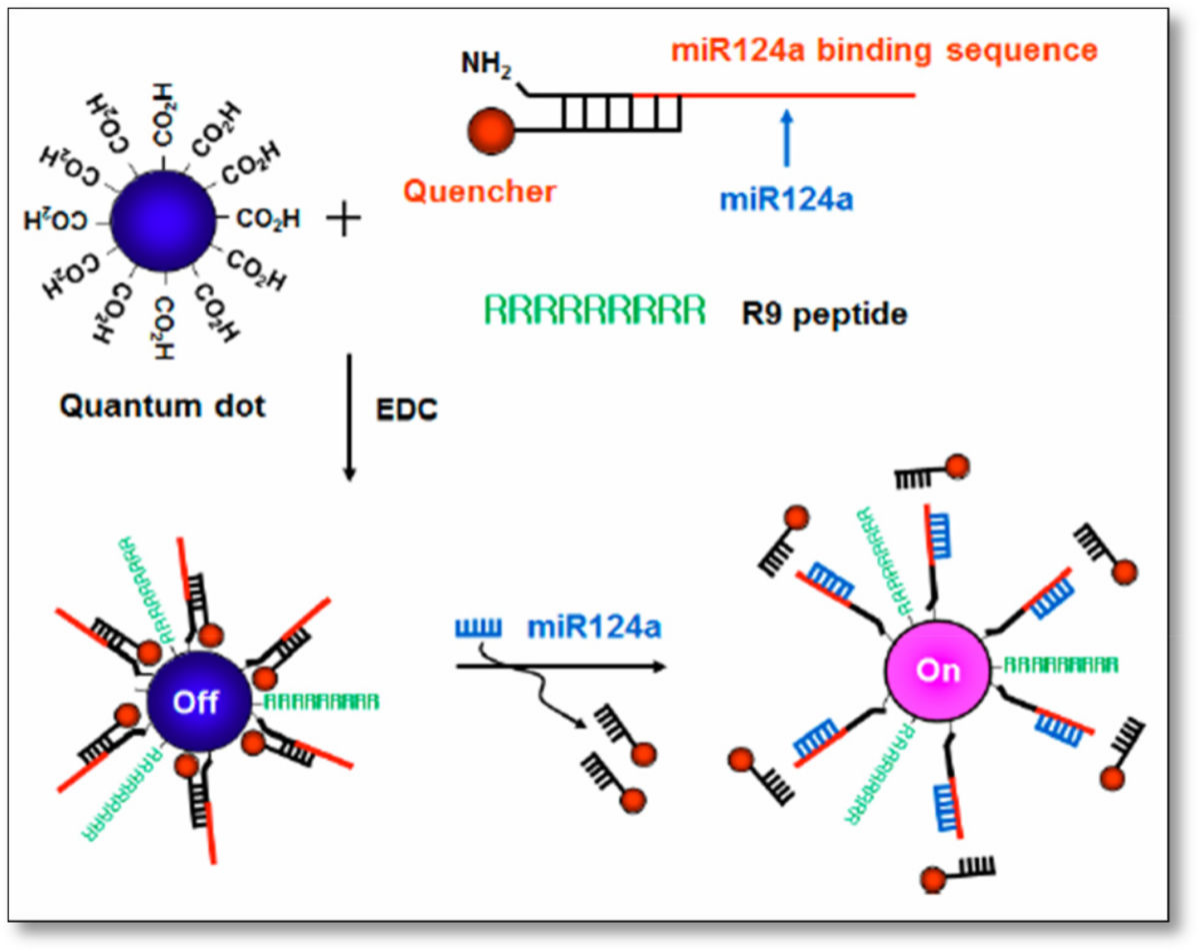
Schematic of R9-QD-mR124a beacons to image mR124a. The oligonucleotide consists of an amine end and a quencher with mR124a recognition sequence. The carboxylated QD was combined with the oligomer and R9 peptide to create R9-QD-mR124a.

Since quantum dots have become interesting fluorophores for biological imaging fields, researchers have developed a new method for the fluorescence imaging of endogenous mRNA using signal-tunable molecular beacon technique based on QDs in living cells. As seen in Figure 5, the group targeted mRNA124a which was observed to have high expression during neuronal development [43]. The beacons, each composed of a quencher on the mRNA124a binding sequence and the R9 peptide, are incorporated with a quantum dots to form R9-QD-mRNA124a beacon. In the absence of mRNA124a, R9-QD-mRNA124a formed a partial duplex beacon that quenches the system, giving no fluorescence signal. With mRNA124a, the mRNA124a binding sequences leave together with the quenchers, leaving a signal of red fluorescence. They concluded that the method could provide critical information on expression during neurogenesis and could be applied to various systems to track cellular developments with different sized QD-based molecular system.

## CONCLUSION

There are many restrictions obtaining multicolor *in vivo* imaging mainly due to overlapping fluorescence emissions. Different approaches have been executed, including the recently developed 3’untranslated region (UTR)-based reported gene imaging system to monitor the expression of patterns of mRNA. However, with this approach, it is difficult to differentiate if the data obtained are from the mRNA expression or from cell death *in vivo* [44–47].

On the other hand, different fluorophores with discernable emissions wavelength must be chosen for multicolor imaging. The complications for the traditional dyes such as organic dyes and QDs are the requirement to use UV or short-wavelength radiation for excitation of the materials and their cytotoxicity in biological environment. Low light penetration depth is observed due to the short wavelength excitation light or low signal-to-noise ratio due to auto-fluorescence. Although fluorescence-based imaging techniques have improved tremendously, there are still rooms to improve the techniques or the probes. It is important to develop more efficient bio-labels to overcome these limitations. NIR probes have gained its momentum the past decades to overcome the shortcomings of the traditional probes when it comes to monitoring the sample with higher depth. Few companies have developed some NIR fluorophores with emission >800 nm, permitting NIR probes to be used together with the original fluorescent organic dye [48]. With the developments of new types of contrast agents together with improvement in electronics and software in equipment, fluorescence-based imaging technique can expand even more. In conjunction of NIR probes, quantum dots have also been an interesting tool not only for imaging field, but for the computing as well as the quantum dot displays for lightning up LCD displays in televisions. Many researches could be done in terms of quantum dots in the field of nanotechnology. Future work such as incorporating the reporter tag into endogenous gene loci using CRISPRCas0 genome editing tools would proof to be quite interesting [49].

The NIR dye, ICG, is already being used to test for liver cancer during surgery, to identify small and unidentifiable liver cancers in real time. ICG has also been used to identify lymph nodes in real time in a small number of breast cancer patients [50]. Even though optical imaging has yet to replace conventional imaging as a form of arthritis tool, OI is a promising diagnostic tool for detecting early onset arthritis.

In summary, this review analysis on the visualization of mRNA with different hybridization techniques. There were many shortcomings since the development of fluorescence-based imaging techniques, but scientists and researchers have all overcome the obstacles and improved both the probes and instruments for a better future. The capability of fluorescence-based imaging techniques is open to new possibilities for better and faster diagnostic of joint diseases in the future.

## Figures and Tables

**Table 1.** Fluorophore labels for molecular beacon probes.

Fluorophore	Alternative Fluorophore	Excitation (nm)	Emission (nm)
TMR	Alexa 546*, Cy3**	555	575
Texas Red	Alexa 594*	585	605
Cy5b	Alexa 647*	650	670

* Alexa fluorophores are available from Invitrogen

** Cyanine dyes are available from Amersham Biosciences

**Table 2.** Quenchers labels for molecular beacon probes.

Quencher	Absorption maximum (nm)
Deep Dark Quencher I*	430
Dabcyl	475
Eclipse**	530
Iowa Black FQ***	532
Black Hole Quencher 1****	534
Black Hole Quencher 2****	580

* Deep Dark Quenchers are available from Eurogentec

** Eclipse quenchers are available from Epoch Biosciences

*** Iowa quenchers are available from Integrated DNA Technologies

**** Black Hole Quenchers are available from Biosearch Technologies
